# Prevention and Treatment of the Diarrhœal Diseases of Infants

**Published:** 1869-09

**Authors:** Stephen Rogers

**Affiliations:** New York


### PREVENTION AND TREATMENT OF THE DIARRHŒAL DISEASES OF INFANTS.

REMARKS MADE BEFORE THE OBSTETRICAL SECTION OF THE NEW YORK ACADEMY OF MEDICINE, MAY 17TH, 1869.

By STEPHEN ROGERS, M.D., New York.

Mr. Chairman:—As a text for the few following remarks, I present a sentence from the address of Sir Thomas Watson, on the occasion of his retiring from the presidency of the Clinical Society of London, because it expresses accurately my own sentiments in reference to the subject before us, and, no doubt, those of many of the Fellows also. "What I deprecate," says Dr. Watson, "what I would fain see altered, what it is one great end of this Society to do away with, is the vagueness of aim, the uncertainty of result, the merely tentative nature of too many of our prescriptions."

In no department of medicine is vagueness of aim, uncertainty of result, and consequently tentative practice, more conspicuous, and, I may add, more disastrous, than in diseases of infants, and especially diarrhœal diseases. We lose our aim, first, by forgetting, or by never knowing, the anatomy and physiology of the infant; and we are forthwith environed by complications and inexplicable phenomena which befog every effort we make.

The digestive apparatus of the infant is, in some respects, like that of the carnivorous animals, arranged for highly animalized and easily assimilable food. This alone should teach us that the pharinaceous and vegetable substances should not enter the diet of young infants. Infants, like animals and like adults, require water; and while their proper food, milk, contains all the water usually demanded, any accident or disease which cuts off the accustomed supply of milk, as well as any circumstance which greatly increases perspiration, such as warm weather, is certain to induce thirst, for which water is the true remedy. The infant intestines, like the adult, are provided with a reservoir for the reception, detention, and absorption of the assimilable fluids. This organ is the *large* intestine, or *colon and cœcum* and the rectum included, and has not like the stomach, and a considerable part of the small intestine, any digestive function. No part of it, therefore, can perform digestion, from the anus to the caput coli—it can only absorb; and substances which are simply in suspension, not in solution, are not appropriated when introduced into this portion of the intestine. Substances, in short, which are not transmitted through membranes by osmosis, are not utilized by the rectum or colon. As an absorbing organ, however, the large intestine is very active. There can be no doubt, I think, that the digestive portions of the intestinal tube of the infant, as well as the absorbing portions, are liable to the same diseases that affect them in the adult; and as diarrhœa is one of the results of disease in both portions, in both adults and infants, we will make our classification upon this understanding. Commencing with the stomach, I will say that diarrhœa *from indigestion* is, perhaps, quite as frequent, if not much more so, in infancy as in adult age. It is very liable to occur in warm weather, to infants both breast and bottle-fed, on account of their taking more milk than the stomach can dispose of, and more than the system requires, the child taking it for thirst instead of for hunger. It is also liable to occur in children bottle-fed on milk too much diluted; the digestive action of the gastric fluids suffering embarrassment by the very great amount of fluid, to say nothing of the grave derangements of the digestive organs, which are produced by insufficiency of nutritive material given in such habitually dilute food. The slow starvation produced by insufficient diet, and by diet which, though sufficient as to quantity, is unsuited as to quality, has many symptoms in common with much of the fatal infantine diarrhœa. This diarrhœa of indigestion is usually ushered in by more or less sudden anorexia, vomiting, thirst, nervous disturbance, and heat of skin, followed in a few hours by diarrhœal discharges, containing more or less undigested food. Unless the cause is repeated, the attack generally subsides with the expulsion of the offending material. It is therefore an exceedingly easy disorder to treat in both the young and old. The treatment, as a rule, need be nothing else than *physiological* and *physical* rest. This is attained by withholding food—a practice readily followed, for the patient does not desire food—quieting thirst by cold water without stint or measure,* keeping the patient still, and, finally, when desire for food may return, to allow it in moderate amounts, and, if it be milk, *undiluted.*

In bottle-fed babies, whose milk has been diluted in the usual manner, from one-half to three-fourths water, nothing can be more striking than the change to undiluted milkman's milk, except, perhaps, when the dilution has been with barley, or other farinaceous decoction. Of all the compounds fruitful of infantile diarrhœa, in our city especially, those by farinaceous decoctions with milk rank first.

Children over six months often desire, and are benefited apparently, by farinaceous food occasionally; but the child should be allowed its choice to take it or not. This cannot be done by mixing it with its milk. All such articles should be given by themselves. The observation of these few plain rules for the treatment of the diarrhœa of indigestion, will be quite sufficient for most cases, medicinal aid being generally unnecessary. To avoid these attacks of diarrhœa of indigestion during our summers, every mother or nurse should be instructed to offer cold water to the infant, whether breast or bottle-fed, before offering it its food, for by so doing, the infant has the opportunity to quench its thirst with water, preserving the unembarrassed energies of the stomach for the digestion of the food.†

* I am ready to stake my reputation as an observer, student of nature, and practitioner, upon the declaration that there is no support in physiology, nor in pathology, nor in practice, for the popular and extensive professional idea that water, to satisfy thirst, can do any infant harm. The idea that it distends the stomach, that it weakens the organ, and prevents digestion, no matter when given, is all clap-trap, without a shadow of foundation in truth. This is a very mild term to use relating to these fostered ideas, if we take into account the untold misery, and even mortality, they annually produce in the diseases of the prima via of infants.

† Much favorable testimony has been furnished the profession, relating to the employment of pepsin in the diarrhœa of indigestion of children and infants. I have not met with cases in which a resort to this agent became necessary, and can therefore offer no experience with it. But as the principle upon which its use is based has a well-known physiological foundation, I am decidedly disposed to adopt it in cases which are not promptly controlled by proper physiological feeding.

Following the intestinal disturbance produced by an attack of diarrhœa of indigestion, the annexed train of symptoms are very often met with:—

Frequent alvine dejections of greenish, very fluid, and fœtid character, frequently containing portions of undigested casein coagulum—if its diet include milk—irritable stomach, and variable appetite, and almost continual thirst. Its discharges may be yellowish, fœtid, and watery, when voided, but become green after a little exposure, generally containing mucus; and there is usually some tenesmus. The child does not have very marked fever, except at varying intervals; emaciation progresses more or less rapidly, and the tongue, as well as the anus, indicate by their redness, enlarged papillæ, and excoriation, a profound disturbance of the alimentary canal.

The case is one of chronic *colitis*, *the usual diarrhœa of infancy.* The colon, as a receiving and absorbing cavity for the excrementitious and alimentary matter poured into it by the small intestine, and by its own excretory glands, refuses to perform its functions; consequently, as fast as material is lodged in it from above, it is hurried on through to the rectum and discharged, not only adding to its own irritability, but not permitting the absorption of much of the alimentary matter provided for it in the canal higher up. During the transit of a fresh supply of such material through the diseased colon, the child often has an intense febrile heat of skin, and not unfrequently convulsions, which terminate life. Both the fact that the morbid changes found after death from diarrhœa in infancy are chiefly in the large intestine, and the phenomena of the disease show conclusively that it is a *colitis* almost exclusively.

When we add to these evidences the results of the *treatment* of colitis, I see no room for a doubt that the usual diarrhœa of infancy, of which so many children die among us annually, is simply colitis. The treatment is clearly to avoid the causes which set up this inflammation, and to lessen the already existing inflammation and irritability. This is accomplished by withholding food as much as possible, keeping the desire for drink satisfied with water, and thus securing physiological rest for the colon. This rest may be more completely effected by calming its pain and irritability by means of anodynes thrown over part of its surface, *viz.:* the rectum. But in this use of anodynes we should never forget that neither the rectum, nor any other part of the large intestine, can digest; that its function is to absorb, and, therefore, nothing should be introduced into it, except solutions, or substances easily soluble in water, and therefore in the moisture of the mucous membrane. Nothing but evil can come from introducing the time-honored *starch*, *gum-water*, *mucilage* of various kinds, oil, albumen, etc., into a diseased and irritable colon or rectum. The watery portions of these preparations are alone absorbed, if retained long enough, and the solid residue is left behind, doing much more harm than good, and often more harm than the combined anodyne does good. The idea that in some forms of inflammatory disease of the large intestine, its usual lubricating covering of mucus is absent, and any of the gummy or mucilaginous substances may in such case be introduced with advantage as a simple protection of the denuded tissue of the mucous membrane, is, to my mind, totally destitute of the support, not only of demonstration, but of probability; and so far as my individual experience may permit me to judge, has not practical support either. Poultices applied to the mucous membrane of the rectum, no matter of what bland substance they be composed, are foreign and excrementitious, and give no rest to the bowel.

Alcoholic solutions, unless largly diluted with water, are liable to irritate, and therefore objectionable. Watery solutions, which leave no solid residum, are clearly the most preferable, and of all preparations, the morphine salts in solution I think best. Where there is not very great irritability of the rectum, the much-used cocoa-butter suppository is a convenient and useful form of introducing the morphine, or other very soluble substances. The warmth of the bowel slowly melts down the mass, allowing the salt to come in contact with the bowel whose moisture dissolves it, and it is then absorbed, while the butter remains as excrement. But the simple watery solution of sulphate of morphine is the least irritating as well as the most active form of anodyne enema. Its dose by the rectum when thus introduced is rather less than by the mouth, and its action is more prompt and more effective to relieve tenesmus and irritability of the large bowel. I have often seen a single injection prevent all movement of the bowels for ten to twelve hours, in cases where the movements before it were almost incessant. Many adjuncts to this treatment will suggest themselves to any educated physician, and I therefore need not mention them here. I will add, however, that I rarely employ any other medication for the diarrhœa of infancy of this degree; and, so far as my observation enables me to judge, much of the favorable results claimed by our practitioners for their favorite prescriptions, such as minute doses of calomel, Dover's powder, ipecac, the sulphite salts, the bromine salts, and the various astringents, alkalies, and anodynes and disinfectants, is due to the concident modification of the diet and care of the child. A diarrhœa, like that just described, of no great severity, having existed for some days, perhaps, suddenly suffers a great increase in the frequency of the movements of the bowels, nausea and vomiting come on, the skin becomes hot, the thirst is urgent, there is more or less extreme restlessness and actual or threatened convulsions. The dejections are, if possible, still more fœtid, watery, and of various colors from black to yellow; they are often streaked or dotted with blood, and the fluid portions sometimes stain reddish the clothes upon which they fall; there is sooner or later mucus intermingled with them, the tenesmus becomes tormenting, the anus red excoriated, and the tumefied mucous membrane of the rectum shows a tendency to descend. This has now become *acute colitis*, *inflammatory diarrhœa* of some authors, or *dysentery* of others. Its treatment does not differ from that already mentioned for colitis, except that it must be conducted with greater energy and watchfulness.

Withhold food as strictly as possible, give cold water *ad libitum*, arrest the pain and irritability of colon by morphine injections, and keep the patient as quiet and cool as practicable, for this is a disease of hot weather.

The use of pure cold water in the irritable stomach of infantile diarrhœa, is theoretically opposed by many practitioners, on the ground that it keeps up the vomiting, as they allege, and furnishes indefinite quantities of fluid to protract the diarrhœal discharges. Practically, I have never seen this theory supported, unless the water were combined with some alimentary substance. It is surprising, however, to see how little milk, or arrowroot, or barley, or any similar substance combined with water, will keep the vomiting and diarrhœa going on to a fatal issue. Pure cold water, on the contrary, will soon arrest vomiting, will give physiological rest to the stomach and intestines, will furnish the much needed fluid to the blood, and thereby calm nervous agitation and afford physical rest and restoration.

As to the treatment of the prolapsus of the mucous membrane of the rectum which we occasionally see follow one of these attacks of colitis, I will add that I have found nothiug of any service which does not arrest the irritability of the part, and the frequent movements of the bowels which attend it. Any agent which secures prolonged repose of the colon and rectum, will cure this condition. The most certain means which I have employed is an injection of the solution of morphine, thrown up immediately after reducing the prolapsed membrane by a coldwater compress and putting the patient to bed. The bowels do not move for twelve to forty eight hours, and recovery of tone and natural condition progresses rapidly. A single application of this kind is generally sufficient, and I have seen very few resist more than two or three. There is still another form of most fatal diarrhœal disease of infancy, presenting the following train of symptons: A mild form of diarrhœa having generally existed for a few days, there suddenly come on vomiting and purging of a copious watery substance, at first containing feculent material, but subsequently an almost pure, opalescent, and nearly odorless fluid, without apparent pain or tenesmus. There is total loss of appetite, great thirst, the surface of the body rapidly becomes cold, the skin shrivelled and moist; in short, a more or less rapid collapse ensues which, as a rule, terminates in fatal convulsions, or anæmic coma, and does so generally within twenty-four hours after the attack. This is the form of diarrhœa, and the only form, in my opinion, to which we should apply the name *Cholera infantum*, and when compared with all the cases of diarrhœal diseases we see, I think the Fellows of the Academy will agree with me in saying, that it composes a small minority of them.

I have no suggestions for its treatment that would not occur to the mind of any physician.

Unquestionably the wisest plan in this, as well as in all the diarrhœal diseases of infancy, is to prevent them if possible. This we may do much to achieve by management of the food. While there is too much evidence to permit us to doubt that, if not a *cause*, dentition at least *attends* a period of development of the digestive apparatus of the infant, during which it is liable to diarrhœal disease, we cannot close our eyes to the fact that very large numbers of our infants die before dentition or any such development commences, before six months, and die of diarrhœal disease. Any extended remarks upon the subject of the diet of infants here I deem uncalled for, and I therefore shall say but little. We, however, all accept the proposition as self-evident, that the best food for the infant is good breast-milk. We are all quite as thoroughly convinced that this is very often not obtainable. Now comes the question as to what is the best substitute for breast-milk. That the milk of some animal should compose the basis of the substitute all, with few insane exceptions, agree. Great numbers of modifications of the milk of the cow—the only available one in this part of the world—have been advocated, chiefly in the degree of its dilution, and the addition of various farinacious substances. But my observations have most thoroughly convinced me that the theoretical dilution of cow's milk, with the view of rendering it similar to the milk of the human female, is an unscientific delusion. It is founded, in the first place, upon the false premises, that our Croton, or other water, is a similar fluid to the watery portions of human milk. Its till further supports itself upon the unfounded assumption, that diluted milk of the cow is more easily digested than the original fluid, on account of the excessive proportion of casein.

There is no means of demonstrating the theory that the addition of water to the milk of the cow renders it more digestible in the infant stomach. But, on the contrary, any one can demonstrate almost any day during our summers, that the labor of the infant's stomach is much easier in the digestion of the best milkman's milk we can obtain here, than it is in the digestion of the usual dilute form, and still less than when diluted with farinacious decoctions. Providence has wisely arranged this matter so that if the milk—the food intended for the infant—be variable as to its constituents, the stomach has the power to digest and more or less completely appropriate them. Hence the milk of the human female, which is often richer in all of its constitutents than many samples of the milk of the cow, is digested, and the child flourishes; and on the contrary, the milk of the cow, which posesses many per cent. more of oil and casein than the average human milk, is easily digested, and the child thrives satisfactorily. The essential points in the whole matter being that the milk given contain nutrient material within a reasonable bulk, sufficient for the nutrition of the child, and that it be given soon enough after leaving the breast or udder to be sweet and good. I avail myself of this opportunity, as I uniformly do of any which presents itself, to denounce the doctrine of dilute cow's milk as infant food, as one destitute of reason and extremely dangerous. If this be true of milk simply diluted, what must be the state of the case when diluted with vegetable and farinaceous substances? For about ten years of my professional life, I have watched this subject closely, having had several children of my own to raise on the milk of the cow. I have yet seen no reason for diluting the milk sold in this city, to make it fit food for the infant at any age. On the contrary, I have often found a necessity for richer milk than could well be obtained here. My experience has satisfied me, that a great part of the difficulty and danger attending the raising of children by hand, as it is called, proceeds from this tinkering of the milk used. I regard the raising of a child with a tolerably good organization as about as easy on the milk of the cow as on the breast. The essential points for the mother or nurse to observe are, that the milk be sweet, that is to say, not soured, that it be warmed to about 100°, that it be taken from the bottle through finely perforated nipples, that the bottle and nipple be kept clean, and finally, *that the child have all it will take.* And here I would repeat the precaution before alluded to, not to give the child milk during the very warm weather of our summers, till water has first been offered to it, else it will often take milk in inordinate quantities simply because it is thirsty, and will thus be overfed and injured. Children at the breast are often injured by this neglect. To those who may possibly regard these views of infant feeding as radical and perhaps dangerous I feel bound to say that I am supported by unquestionably competent authority. While writing these pages, I had the exteme gratification of receiving the pamphlet paper on "Food for Infants," lately read before the Medical Society of the State of Pennsylvania, by Dr. Hiram Corson, of that State. The Dr. has extended his observations through more than thirty years, and they have obviously been well and carefully conducted. He says, "I feel quite certain that it is almost as easy to raise children by hand, if they have an abundant supply of good undiluted cow's milk, as it is by the breast." And he repeats this expression of belief, adding, with great propriety and force—as his observations are conducted in the country and villages where good milk is easily obtainable—"if, then, in the country, where the milk is good, the child should have all it will take undiluted, how very important that no water should be added to the milk brought to cities by milkmen. It is not too much to say that before it reaches the citizen's door it is only two-thirds milk." He details the symptoms he has often seen presented by infants fed, but only half nourished, on dilute milk, and those descriptions accurately apply to great numbers of the diarrhœal disease we see here. His experience leads him to conclude that thousands of infants who die annually of these diseases, really "die from want of food." "They are starved to death," says he, speaking to the profession, "and we are not blameless." Again he says: "Little children not only need plenty of good food, but, even those who are fed at a full breast, also need occasionally a little cool water as drink."

This sentiment I am delighted to see as thus generally stated, but in the warm weather of our summers it is especially applicable. Dr. Corson very justly wonders that these facts have not more generally impressed themselves upon physicians, and that as a consequence the public teachers with few exceptions, and the text-book of to-day, are promulgating the same doctrine and giving the same rules that they have for the last hundred years, changing, if at all, for the worse, for higher dilutions, and in the face of the frightful fact that infant mortality is increasing rather than diminishing. He very properly suggests that we try a change, which can hardly be for the worse. This is very grateful support to the sentiments I uttered many months ago, in the pages of the *Record* of Oct. 1st, 1868, p. 341. With this care to properly feed infants, much, very much, can be done to prevent their diarrhœal diseases, especially diarrhœas from indigestion; and with these simple measures for the treatment of chronic and acute colitis, vastly better results, I am convinced, may be obtained. The comparative experience of many physicians, male and female, in the city and in the country, confirms me in this belief, which I formed from an extensive public practice, added to that on my own family, and in private. —*N. Y. Med. Record.*

